# The Longitudinal Mediating Role of Academic Buoyancy Between Academic Self-Efficacy and Academic Burnout Among Junior High School Students: A Cross-Lagged Study

**DOI:** 10.3390/bs15111480

**Published:** 2025-10-30

**Authors:** Licong Ye, Yongchun Xie, Baojuan Ye

**Affiliations:** 1 Public Teaching Department, Fuzhou Medical University, Fuzhou 344000, China; 2 School of Psychology, Jiangxi Normal University, Nanchang 330022, China; xyc20010317@163.com

**Keywords:** academic self-efficacy, academic buoyancy, academic burnout, mediation effect

## Abstract

This study aims to explore the longitudinal relationship between academic self-efficacy, academic buoyancy, and academic burnout among junior high school students and to reveal the potential mediating role of academic buoyancy. Using cluster sampling, a longitudinal study was conducted on 906 students (mean age = 12.48, 53.3% male) in grades 7 to 9, with three follow-up assessments conducted at four-month intervals. The assessment tools included the Academic Self-Efficacy Questionnaire, the Academic Buoyancy Scale, and the Academic Burnout Questionnaire. The results indicated the following: (1) academic self-efficacy and academic buoyancy exhibit a bidirectional relationship, meaning that academic self-efficacy is associated with increases in academic buoyancy levels four months later, and academic buoyancy also is associated with increases in academic self-efficacy four months later; (2) academic buoyancy is associated with decreases in academic burnout four months later, and academic burnout also is associated with decreases in academic buoyancy four months later; (3) academic self-efficacy is indirectly associated with decreases in academic burnout through the mediating effect of academic buoyancy. Research implications: In educational practice, a focus should be on enhancing students’ academic self-efficacy while effectively reducing academic burnout among junior high school students by fostering the psychological resource of academic buoyancy.

## 1. Introduction

Academic burnout—a state of emotional exhaustion, cynicism, and reduced efficacy resulting from chronic academic stress—poses a severe threat to adolescent well-being globally (Maslach et al., 2001) This issue is particularly acute during the junior high school years, a period of significant bio-psycho-social changes exacerbated by intense academic competition (Demkowicz et al., 2024). In the context of Chinese exam-oriented education, the pressure from high-stakes testing has made burnout increasingly common, undermining learning efficiency and leading to adverse mental health outcomes such as depression and anxiety (Fiorilli et al., 2017; Gao, 2023; Wu et al., 2007). Therefore, identifying malleable psychological resources that can buffer against chronic academic stress is of paramount importance. This pursuit is powerfully guided by the Conservation of Resources (COR) theory (Hobfoll, 1989, 2001). COR theory posits that individuals strive to obtain, retain, and protect their valued resources, and psychological stress occurs when these resources are threatened, lost, or when investment of resources fails to yield adequate gains. From this perspective, academic burnout can be understood as a state of chronic resource depletion.

In this pursuit, academic self-efficacy has been established as a critical protective factor, with robust evidence showing its significant negative correlation with burnout (Charkhabi et al., 2013; C. Chen et al., 2023). This relationship is often framed through Conservation of Resources Theory, which posits self-efficacy as a key personal resource that prevents psychological depletion (Hobfoll, 1989). However, the existing literature is constrained by two key limitations. First, its reliance on cross-sectional data leaves the longitudinal and potentially bidirectional dynamics between these constructs inadequately examined (Cengiz & Peker, 2024; Schaufeli et al., 2002). Second, and more critically, a predominant focus on direct effects has overlooked the underlying mediating mechanisms that explain how self-efficacy translates into reduced burnout.

To address these gaps, this study introduces academic buoyancy—students’ capacity to effectively navigate daily academic adversities—as a pivotal mediator (Martin & Marsh, 2008). Research confirms that buoyancy is negatively associated with burnout (Lakkavaara et al., 2024; Putwain et al., 2020) and shares a close, likely bidirectional relationship with self-efficacy, as confident students engage in more adaptive coping, and successful coping, in turn, boosts confidence through “micro-success” experiences (Kang et al., 2024; Weißenfels et al., 2023). This proposed pathway is conceptually aligned with Social Cognitive Theory (Bandura, 1989), which elucidates the dynamic interaction between cognition (self-efficacy), behavior (buoyancy), and environment, while Conservation of Resources Theory explains the resource investment and protection process. Consequently, this longitudinal study investigates the bidirectional relationships between self-efficacy, buoyancy, and burnout, and tests the mediating role of academic buoyancy among Chinese junior high school students.

By integrating two major psychological theories within a longitudinal design, this research aims to overcome prior methodological limitations and provide a nuanced understanding of the protective mechanism against burnout. The findings are expected to offer significant theoretical insights and a precise, actionable target for educational interventions aimed at fostering student resilience and well-being.

### 1.1. Academic Self-Efficacy: A Foundational Motivational Belief

Academic self-efficacy, originating from Bandura’s (1977, 1989) social cognitive theory, refers to an individual’s conviction in their capabilities to organize and execute the courses of action required to manage prospective academic situations. In the educational domain, this construct is often conceptualized as comprising two primary dimensions: academic ability self-efficacy, which pertains to one’s belief in their competence to successfully accomplish academic tasks and achieve high performance, and academic behavior self-efficacy, which involves the expectations and evaluations related to the learning strategies and methods employed to attain those goals (Liang, 2000).

A robust body of empirical evidence underscores the pivotal role of academic self-efficacy as a protective factor against student maladjustment. Specifically, research has consistently demonstrated its significant negative association with academic burnout. Students with heightened self-efficacy are less likely to experience the core symptoms of burnout, namely emotional exhaustion, academic cynicism, and a reduced sense of accomplishment (Charkhabi et al., 2013; C. Chen et al., 2023; Gao, 2023). This protective mechanism can be effectively interpreted through the lens of Conservation of Resources (COR) theory (Hobfoll, 1989). From this perspective, academic self-efficacy constitutes a vital personal resource. Students rich in this resource are more capable of deploying effective coping strategies when facing academic demands, thereby preventing the chronic depletion of their psychological energy that characterizes burnout (Chaudhry & Chhajer, 2023; Schaufeli et al., 2002). Furthermore, from the COR theory perspective, such individuals are positioned to invest their self-efficacy resources to acquire and build other protective resources (Hobfoll, 2001). Conversely, students with low self-efficacy, lacking confidence in their ability to cope with academic challenges, are more susceptible to entering a vicious cycle where initial burnout further erodes their self-efficacy, leading to more severe burnout (Bandura, 1977). COR theory conceptualizes this dynamic as a loss spiral, wherein initial resource loss (e.g., academic setbacks) amplifies vulnerability, leading to further depletion of key resources and more severe states of exhaustion (Hobfoll, 1989, 2001).

However, a critical analysis of the extant literature reveals a notable methodological limitation. While the negative correlation is well-established, the majority of supporting studies are grounded in cross-sectional data (Charkhabi et al., 2013; Rohmani & Andriani, 2021, 2021). This design inherently precludes a robust examination of the longitudinal and potentially bidirectional nature of the relationship between self-efficacy and burnout. Although the possibility of reciprocity has been theoretically suggested and sporadically identified (Cengiz & Peker, 2024; Schaufeli et al., 2002), the precise temporal dynamics—how these two constructs influence and reinforce each other over time, particularly among junior high school students—remain inadequately examined. This gap necessitates a longitudinal design to unravel the causal precedence and enduring interplay between these crucial variables. Therefore, this study will first examine the bidirectional longitudinal relationship between academic self-efficacy and academic burnout (Hypothesis 1).

### 1.2. Academic Buoyancy: Navigating Everyday Academic Adversity

In contrast to broader conceptions of resilience that focus on overcoming significant adversity, academic buoyancy is conceptualized as students’ capacity to successfully navigate the typical “ups and downs” of everyday academic life (Martin & Marsh, 2008). This construct manifests in students’ adaptive responses to routine challenges such as poor grades, workload pressure, and negative feedback, encompassing psychological components like stress coping, frustration tolerance, and setback recovery (Martin & Marsh, 2008; Salimzadeh et al., 2021). As such, academic buoyancy itself constitutes a critical personal resource within the COR framework, one that can be drawn upon to navigate demands and prevent resource depletion.

A growing body of international research positions academic buoyancy as a critical protective factor against academic burnout. Empirical findings consistently demonstrate a significant negative relationship between the two constructs. For instance, a longitudinal study by Putwain et al. (2020) found that baseline levels of buoyancy were significantly linked to lower subsequent academic burnout among secondary school students. This buffering effect is attributed to buoyant students’ propensity to employ proactive coping strategies (e.g., problem-solving and emotion regulation) when faced with academic setbacks, which interrupts the accumulation of chronic stress that leads to burnout (Lakkavaara et al., 2024; Teixeira et al., 2022; Vinter, 2021). COR theory reframes this process as the successful investment of buoyancy resources to prevent the loss of more fundamental resources, thereby shielding the individual from the state of exhaustion that characterizes burnout. In some studies, buoyancy has even been identified as the strongest protective factor against burnout symptoms (Vinter, 2021).

Crucially, academic buoyancy does not operate in isolation but is dynamically linked with academic self-efficacy. Research indicates a likely bidirectional relationship between them. On one hand, students with high academic self-efficacy possess greater confidence in their ability to handle challenges, making them more likely to initiate and persist in buoyant, adaptive coping behaviors (Kang et al., 2024; Weißenfels et al., 2023). On the other hand, the experience of successfully bouncing back from daily setbacks provides students with positive “micro-success” experiences, which in turn serve to strengthen their belief in their academic capabilities, thereby enhancing their self-efficacy (Collie et al., 2015; Putwain et al., 2020).

This reciprocal relationship suggests that self-efficacy and buoyancy may function as a resource caravan (Hobfoll, 2001), where these resources tend to co-occur and mutually reinforce one another over time. Notwithstanding these important findings, the existing literature exhibits a significant gap. While the direct protective effect of buoyancy against burnout is established, and its correlation with self-efficacy is recognized, research has seldom positioned buoyancy within a longitudinal mediation framework. The question of whether academic buoyancy serves as the key mechanism that translates the benefits of academic self-efficacy into long-term protection against burnout remains largely unexplored. This represents a critical next step in understanding the functional relationships between these psychological resources (Cengiz & Peker, 2024). Consequently, the present research will also investigate the reciprocal associations between academic buoyancy and academic burnout, as well as between academic self-efficacy and academic buoyancy (Hypotheses 2 and 3, respectively).

### 1.3. The Interplay of Self-Efficacy, Buoyancy, and Burnout: Toward an Integrated Model

Building upon the established relationships between the constructs, an integrated model that examines academic buoyancy as a mediator between self-efficacy and burnout offers a more nuanced explanation of their interplay. This integrated model is undergirded by two complementary theoretical frameworks. Social Cognitive Theory outlines the mediational pathway, specifying how the cognitive belief of academic self-efficacy promotes the adaptive behaviors encompassed by academic buoyancy to achieve the outcome of reduced burnout (Bandura, 1989). According to COR theory (Hobfoll, 1989, 2001), which provides the motivational principle, individuals invest key resources such as self-efficacy to gain and protect other resources like buoyancy, thereby forming a resource caravan. This caravan of resources effectively shields against resource loss, which constitutes the core of burnout. Furthermore, the COR theory concept of loss spirals predicts the observed reciprocity, wherein burnout as a state of resource depletion can, in turn, erode subsequent levels of self-efficacy and buoyancy.

Empirical evidence lends preliminary support to this integrated perspective. The well-documented negative effect of self-efficacy on burnout (Charkhabi et al., 2013; Gao, 2023) and the positive link between self-efficacy and buoyancy (Kang et al., 2024; Weißenfels et al., 2023) establish the first part of the chain. Furthermore, the strong negative association between buoyancy and burnout is equally well-established (Lakkavaara et al., 2024; Putwain et al., 2020). Crucially, the potential for reciprocal influences must be considered. For instance, there is evidence that burnout shows a negative association with subsequent self-efficacy and buoyancy, suggesting a risk of a vicious cycle where depleted resources hinder the development of protective factors (Cengiz & Peker, 2024; Pham Thi & Duong, 2024; Schaufeli et al., 2002).

Despite these valuable insights, a critical synthesis of the literature reveals a definitive gap. While the pairwise relationships are supported, studies have rarely investigated the dynamic, longitudinal relationships among all three constructs simultaneously within a single, comprehensive model. The core proposition of this study is that academic buoyancy serves as the central mechanism through which self-efficacy exerts its longitudinal influence on burnout. Furthermore, this mediating process is likely embedded within a network of reciprocal effects, including the potential for burnout to erode earlier resources. This dynamic model remains largely untested. Therefore, research that employs a longitudinal cross-lagged design to dissect these complex temporal and causal relationships is necessary to move beyond correlation and toward a clearer understanding of the underlying psychological processes (Cengiz & Peker, 2024). Guided by this integrated model, the core aim of this study is to test the proposition that academic buoyancy serves as the key mediating mechanism through which academic self-efficacy exerts its longitudinal influence on reduced academic burnout (Hypothesis 4).

### 1.4. Hypotheses of the Present Study

Based on the theoretical integration and literature review presented above, this study proposes and aims to test the following hypotheses regarding the longitudinal relationships among academic self-efficacy, academic buoyancy, and academic burnout:

**H1.**  *There is a bidirectional relationship between academic self-efficacy and academic burnout among junior high school students.*

**H2.**  *There is a bidirectional relationship between academic buoyancy and academic burnout among junior high school students.*

**H3.**  *There is a bidirectional relationship between academic self-efficacy and academic buoyancy.*

**H4.**  *Academic self-efficacy is indirectly linked to academic burnout via its association with academic buoyancy.*

## 2. Materials and Methods

### 2.1. Participants

A longitudinal study was conducted using a cluster sampling method involving 906 students from grades 7 to 9. The follow-up intervals for the study were four months apart, with three measurement points. Among the participants, 483 were male (53.3%) and 423 were female (46.7%), with an average age of 12.48 years (SD = 0.96). The sample included students from all three grades: 328 (36.2%) in the first grade, 298 (32.9%) in the second grade, and 280 (30.9%) in the third grade.

Regarding their place of origin, 682 students (75.3%) were from urban areas, while 244 students (26.3%) were from rural areas. In terms of family structure, 156 students (17.2%) were only children, whereas 750 students (82.8%) were not.

The first survey questionnaire (T1) was distributed in October 2023, the second questionnaire (T2) in February 2024, and the third questionnaire (T3) in June 2024, maintaining a four-month interval between each measurement point. Due to the nature of the longitudinal study, some missing data were unavoidable. Participants were selected based on the criteria that they completed all three tests, showed no evidence of patterned responding, and had less than 5% missing values. Ultimately, 906 participants met these criteria and were included in the analysis. For the minimal random missing values that remained in the dataset of these 906 participants, we employed the Expectation-Maximization (EM) algorithm in SPSS to generate a single complete dataset for all subsequent analyses.

### 2.2. Research Tools

#### 2.2.1. Academic Self-Efficacy Questionnaire

The Academic Self-Efficacy Questionnaire developed by Liang (2000) was used in this study. This scale consists of two dimensions: academic ability self-efficacy and academic behavior self-efficacy, with a total of 22 items. The scale employs a 5-point Likert scale (1 = completely disagree, 5 = completely agree), with higher scores indicating higher levels of academic self-efficacy. In this study, the Cronbach’s α coefficients of this measurement tool at three time points were 0.904 (T1), 0.921 (T2), and 0.930 (T3), respectively, indicating that the questionnaire had good internal consistency.

#### 2.2.2. Academic Buoyancy Scale

The Academic Buoyancy Scale developed by Martin and Marsh (2008) was employed. The scale consists of four items that assess whether students can effectively cope with daily academic obstacles in terms of stress coping, frustration coping, stress tolerance, and negative academic feedback. After localization and revision (Sun, 2009), the scale uses a 5-point Likert scale (1 = completely disagree, 5 = completely agree), with higher scores indicating higher levels of academic buoyancy. In this study, the Cronbach’s α coefficients of this measurement tool at three time points were 0.776 (T1), 0.825 (T2), and 0.859 (T3), indicating good internal consistency of the questionnaire.

#### 2.2.3. Academic Burnout Scale

The questionnaire developed by Wu et al. (2007) was used to measure academic burnout among junior high school students. The scale consists of 16 items covering three dimensions: physical and mental exhaustion, academic alienation, and low sense of achievement. The scale uses a 5-point Likert scale (1 = completely disagree, 5 = completely agree), with higher scores indicating more severe academic burnout. In this study, the Cronbach’s α coefficients of this measurement tool at three time points were 0.795 (T1), 0.819 (T2), and 0.812 (T3), respectively, indicating that the questionnaire had good internal consistency.

### 2.3. Research Procedure

A paper-based questionnaire survey method was employed, and informed consent was obtained from the school, students, and parents before conducting the test. Master’s and doctoral students who had trained in the standardized data collection procedure acted as examiners. After explaining the study’s purpose, the students were organized by class and asked to complete the questionnaire in their classroom. The examiners provided clear instructions and precautions, emphasized the importance of honest responses, and assured the students that their information would be kept confidential and used only for research purposes. The entire testing process took approximately 40 min.

### 2.4. Data Analysis

This study used SPSS 26.0 and Mplus 7.4 for data analysis. The single complete dataset generated via the Expectation-Maximization (EM) algorithm in SPSS (as detailed in Section 2.1) was used for all subsequent analyses, including the cross-lagged model estimation in Mplus. Specific methods included: Harman’s single-factor test to assess common method bias; descriptive statistics to present data characteristics; t-tests and analysis of variance to compare differences between groups; Pearson correlation analysis to explore variable correlations. Longitudinal equivalence tests were conducted on the three variables at three time points to ensure the stability of the measurement tools across time. A cross-lagged model was constructed to analyze the bidirectional interaction mechanism of the three variables. Finally, the significance of the mediating path of academic buoyancy was tested using the Bootstrap repeated sampling technique (5000 samples, 95% confidence interval).

## 3. Results

### 3.1. Measurement Equivalence Test

To explore the dynamic interactions between variables, this study uses a cross-lagged model to analyze the self-stability of each variable and its interactions. According to Byrne (2012), longitudinal data analysis must first ensure the cross-temporal equivalence of measurement tools to guarantee the reliability and comparability of data results. Based on this, this study used confirmatory factor analysis (CFA) to test the equivalence of the measurement tools at three time points: T1, T2, and T3. In the model construction process, single-dimensional variables were used as latent variable indicators based on their items, while multi-dimensional variables were used as latent variable indicators based on their dimensions. The study conducted configurational equivalence, weak equivalence, and strong equivalence tests on academic self-efficacy, academic buoyancy, and academic burnout in sequence. Given that the chi-square statistic is sensitive to sample size, this study followed F. F. Chen’s (2007) recommendation and used the changes in CFI and RMSEA (ΔCFI ≤ 0.01 and ΔRMSEA ≤ 0.015) as the criteria for judging model fitting differences. The results of the data analysis are shown in Table 1. Academic self-efficacy, academic buoyancy, and academic burnout all met the requirements for strong equivalence, and the fitting indices of each model met the statistical standards, indicating that the factor structure was equivalent across time.

### 3.2. Descriptive Statistics and Correlation Analysis of Variables

The correlation coefficients between variables are shown in Table 2. There was a significant positive correlation between academic self-efficacy and academic buoyancy in the three measurements, and a significant negative correlation with academic burnout. There was also a significant negative correlation between academic buoyancy and academic burnout.

Based on the guidelines for interpreting correlation coefficients (Cohen, 1992), where |*r*| ≥ 0.1 is small, |*r*| ≥ 0.3 is medium, and |*r*| ≥ 0.5 is large, the stabilities and concurrent correlations in our study demonstrated medium to large effect sizes. As shown in Table 2, the longitudinal stabilities (e.g., T1 and T2 academic self-efficacy, *r* = 0.59) were large. Similarly, concurrent correlations were substantial, such as the large association between T1 self-efficacy and T1 buoyancy (*r* = 0.62) and the medium negative correlation between T1 buoyancy and T1 burnout (*r* = −0.40). These findings confirm that the variables are not only statistically related but also share meaningful associations, providing a solid foundation for the cross-lagged analyses.

### 3.3. The Longitudinal Relationship Between Academic Burnout, Academic Self-Efficacy, and Academic Buoyancy Among Junior High School Students

**Figure 1:**
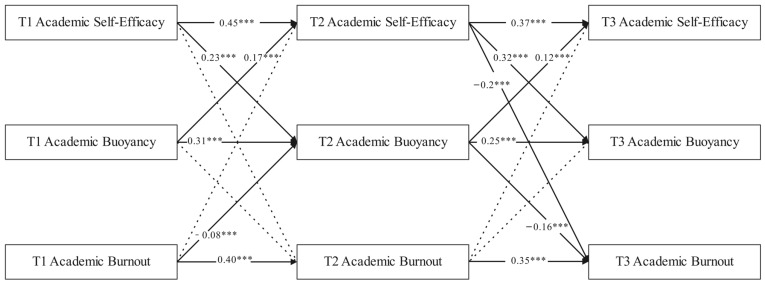
Cross-lagged model diagram of academic self-efficacy, academic buoyancy, and academic burnout. Note: The model presents unstandardized coefficients. Solid lines indicate significant paths. Solid lines indicate significant paths (*p* < 0.05), while dashed lines indicate non-significant paths. *** *p* < 0.001. The use of unstandardized coefficients is preferred as they reflect the relationships among variables in their original metrics, facilitating a direct interpretation of effect magnitude (McNeish & Hamaker, 2020; Schuurman et al., 2016).

In presenting the model results, we primarily report unstandardized coefficients. This decision is grounded in methodological recommendations that unstandardized coefficients are preferable when variables are measured on meaningful scales, as they allow for a direct interpretation of the effect in the original units of measurement and are not conflated with between-person differences in variance (McNeish & Hamaker, 2020; Schuurman et al., 2016). As shown in Figure 1, a cross-lagged model was constructed based on correlation analysis, and model fitting tests were conducted. The overall model fitting Index was good: *CFI* = 0.983, *TLI* = 0.921, *RMSEA* = 0.074, *SRMR* = 0.029. The direct temporal relationship between academic self-efficacy and academic burnout was only partially significant, with T2 academic self-efficacy being significantly negatively associated with T3 academic burnout (*γ* = −0.20, *p* < 0.001), while the remaining paths did not reach significance (*p* > 0.05).

T1 academic buoyancy was not significantly associated with T2 academic burnout (*γ* = −0.04, *p* > 0.05), but T2 academic buoyancy was significantly negatively associated with T3 academic burnout (*γ =* −0.16, *p* < 0.001). In addition, T1 academic burnout was significantly negatively associated with T2 academic buoyancy (*γ* = −0.08, *p* < 0.01), while the effect of T2 academic burnout on T3 academic buoyancy did not reach a significant level (*γ* = −0.04, *p* > 0.05).

The level of academic self-efficacy in stage T1 is positively associated with the development of academic buoyancy in stage T2 (*γ* = 0.23, *p* < 0.001), and academic self-efficacy in stage T2 is positively associated with academic buoyancy in stage T3 (*γ* = 0.32, *p* < 0.001). At the same time, academic buoyancy also demonstrated a significant association with effect on academic self-efficacy. Academic buoyancy in T1 was positively associated with academic self-efficacy in T2 (*γ* = 0.17, *p* < 0.001), and academic buoyancy in T2 was positively associated with academic self-efficacy in T3 (*γ* = 0.12, *p* < 0.001). The results of the cross-lagged model showed that academic self-efficacy and academic buoyancy had a bidirectional relationship with temporal precedence.

The mediation effect analysis showed that academic self-efficacy indirectly affected academic burnout through academic buoyancy (mediation effect was −0.026, *p* < 0.05), while academic burnout also indirectly affected academic self-efficacy through academic buoyancy (mediation effect was −0.016, *p* < 0.05). Academic buoyancy has a significant longitudinal mediating effect between academic self-efficacy and academic burnout.

## 4. Discussion

### 4.1. Academic Self-Efficacy and Academic Burnout Among Junior High School Students

Regarding Hypothesis 1, consistent with previous studies (Charkhabi et al., 2013; Gao, 2023), this study found a negative correlation between academic self-efficacy and academic burnout among junior high school students, meaning that higher levels of academic self-efficacy are associated with lower levels of academic burnout. This result is also consistent with the dynamic model of psychological resources proposed by the conservation of resources theory (Hobfoll, 1989), which states that academic burnout, as a state of depletion of psychological resources, weakens junior high school students’ academic self-efficacy, and low academic self-efficacy further exacerbates the degree of academic burnout. Unlike previous studies (Cengiz & Peker, 2024), we did not find a significant direct effect of burnout on subsequent self-efficacy. This discrepancy is likely due to model specification: by including academic buoyancy, our model reveals that burnout’s effect is primarily transmitted indirectly through the depletion of this resource. Therefore, the apparent contradiction is resolved by identifying a more precise mechanism, highlighting the critical role of academic buoyancy.

### 4.2. Academic Burnout and Academic Buoyancy Among Junior High School Students

In support of Hypothesis 2, and consistent with previous studies (Lakkavaara et al., 2024; Vinter, 2021), this study found evidence of a longitudinal, albeit asymmetric, relationship between academic buoyancy and academic burnout among junior high school students; that is, academic burnout was negatively associated with academic buoyancy, and conversely, academic buoyancy was negatively associated with academic burnout. Academic burnout is mainly manifested as emotional exhaustion, loss of interest in learning, and a significant decline in a sense of accomplishment (Maslach et al., 2001). When junior high school students feel exhausted and unmotivated, they may find it more difficult to actively respond to academic challenges, leading to a decline in academic buoyancy. This result is consistent with the effort-reward imbalance model (Siegrist, 1996), in which academic buoyancy mediates the relationship between effort-reward imbalance and academic burnout and can effectively reduce academic burnout. Furthermore, the specific longitudinal pattern—where T1 buoyancy failed to predict T2 burnout, but T2 buoyancy significantly predicted T3 burnout—can be explained by the escalating academic context. Drawing on Conservation of Resources Theory (Hobfoll, 1989), we posit that the protective role of academic buoyancy becomes critical only when cumulative demands push students’ resource pools toward a depletion threshold. The initial period (T1–T2) may not have crossed this threshold, rendering early buoyancy less decisive. However, by T2, sustained stress creates a state of heightened vulnerability, making existing buoyancy resources essential for preventing burnout at T3.

### 4.3. Academic Self-Efficacy and Academic Buoyancy

Confirming Hypothesis 3, and consistent with previous studies (Kang et al., 2024; Weißenfels et al., 2023), this study identified a bidirectional relationship between academic self-efficacy and academic buoyancy; that is, academic self-efficacy was positively associated with academic buoyancy, and vice versa. Students with heightened academic self-efficacy display increased confidence in the face of academic challenges and adopt more proactive coping strategies when encountering adversity, thereby enhancing their academic buoyancy. This increased buoyancy, in turn, leads them to pursue more challenging goals and persist in the face of difficulties, creating a positive reinforcing feedback loop. (Kang et al., 2024; Weißenfels et al., 2023). Academic buoyancy feeds back into self-efficacy through the accumulation of “micro-success” experiences. Academically buoyant junior high school students face challenges with greater confidence. By successfully regulating their emotional state in response to daily setbacks, they develop the positive belief that “I can cope with difficulties” thereby strengthening their academic self-efficacy (Putwain et al., 2020).

### 4.4. Academic Burnout, Academic Self-Efficacy, and Academic Buoyancy Among Junior High School Students: The Mediating Role of Academic Buoyancy

The results of this study show support for Hypothesis 4, indicating that academic buoyancy plays a significant partial longitudinal mediating role between academic self-efficacy and academic burnout. This mediating role can be understood by integrating social cognitive theory (Bandura, 1989) and conservation of resources theory (Hobfoll, 1989), by illustrating two key psychological mechanisms.

First, from the perspective of social cognitive theory, the mechanism is one of “Proactive Coping Deployment.” Students with high academic self-efficacy possess stronger beliefs in their capabilities to manage academic challenges. This confident cognition directly influences their behavior, predisposing them to proactively deploy adaptive strategies—such as problem-solving, seeking help, and effective emotion regulation—when confronted with daily academic setbacks (Martin & Marsh, 2008). This constellation of adaptive behaviors is the very essence of academic buoyancy. In other words, self-efficacy fuels the buoyant behaviors that help students navigate academic pressures before they escalate into chronic stress.

Second, conservation of resources theory elucidates the “Resource Caravan and Protection Mechanism.” According to COR theory, individuals strive to obtain, retain, and protect their valuable psychological resources. Academic self-efficacy is a critical personal resource. This resource invests into and builds another key resource—academic buoyancy—creating a “resource caravan” (Hobfoll, 2001). Academically buoyant students, through their effective coping, successfully protect their existing resources (e.g., cognitive capacity, emotional energy) from excessive depletion by routine academic stressors. By preventing this resource drain, buoyancy directly shields students from the core symptoms of burnout, which are characterized by exhaustive resource loss (emotional exhaustion), detachment from the resource-investing context (academic cynicism), and a diminished sense of resource gain (reduced accomplishment).

Therefore, the internal mechanism is not merely a statistical chain but a dynamic psychological process: Academic self-efficacy (a key cognitive resource) drives the enactment of academically buoyant behaviors (proactive coping), which in turn function as a protective shield, conserving the individual’s overall resource pool and thereby preventing the development of academic burnout. Academic burnout can induce a variety of psychological adaptation problems, including anxiety disorders, depression, and other adverse psychological reactions (Liu et al., 2024). These adverse psychological states damage students’ mental health and interfere with cognitive functions, thereby weakening their capacity to cope with and adapt to challenges (Nogueira & Sequeira, 2024). Reduced academic burnout frees up time and energy, which students can then actively engage in learning (C. Chen et al., 2023) and deploy effective strategies to solve problems, thereby enhancing their academic buoyancy. High levels of buoyancy, in turn, facilitate coping with daily academic setbacks and promote positive emotional experiences—such as pleasure, satisfaction, and accomplishment—in learning contexts (Martin et al., 2013). These emotions boost students’ self-confidence, motivation, and self-efficacy. In summary, improving academic buoyancy represents a viable approach to reducing academic burnout among junior high school students.

The magnitudes of the specific indirect effects were modest, at −0.026 from self-efficacy to burnout and −0.016 from burnout to self-efficacy. In line with methodological guidance for longitudinal research (Shrout & Bolger, 2002), even effects of this limited size can delineate a meaningful mediating pathway when they are statistically reliable. Thus, academic buoyancy is confirmed to function as a significant, albeit modest, longitudinal mediator in the reciprocal relationship between self-efficacy and burnout.

### 4.5. Research Limitations and Implications

While this study focused on individual variables, Ecological Systems Theory posits that environmental factors also significantly influence individual development. Therefore, future research should integrate environmental variables (e.g., classroom climate, teacher support, family environment) to provide a more comprehensive understanding of the causes of academic burnout. Additionally, employing multi-informant (e.g., teacher reports) or behavioral measures would help overcome the limitations of relying solely on self-report data and provide more robust evidence for the relationships observed. It would also be crucial to formally test the demographic variables we collected (gender, grade level, urban-rural residence) as moderators, to determine whether the observed relationships hold across different subgroups. Furthermore, future studies should expand the scope of sampling to include more representative and diverse populations, which would help elucidate the characteristics and differences in academic burnout across various groups.

Despite its limitations, this study elucidates one of internal mechanism through which academic self-efficacy influences academic burnout via the mediating role of academic buoyancy, offering valuable insights for educational practice. Theoretically, this study enriches the academic burnout literature by introducing a positive psychology perspective, thereby expanding the theoretical foundation and providing a scientific basis for mitigation strategies. Practically, this research contributes to the field in three key aspects: First, by thoroughly analyzing the current state of academic burnout, these findings call for targeted interventions across multiple systems: for educators, to develop programs that directly build students’ academic buoyancy; for parents, to foster a home environment that supports self-efficacy through autonomy and mastery experiences; and for policymakers, to allocate resources for school-based mental health initiatives that address burnout proactively. Second, by examining the relationships between academic self-efficacy, academic buoyancy, and academic burnout, it reveals underlying psychological mechanisms and offers an evidence-based foundation for developing targeted educational interventions. Third, clarifying the mediating role of academic buoyancy can lead to the creation of practical strategies to reduce burnout by enhancing students’ adaptive capacity. Developing and implementing structured academic buoyancy training programs within school mental health curricula could help students establish resilient learning mechanisms and foster their psychological well-being. Fourth, despite attrition analysis indicating data were missing completely at random, the reduction in sample size from initial recruitment to final analysis should be noted as a potential limitation for the generalizability of the findings (Gustavson et al., 2012).

### 4.6. The Role of Cultural Context

The present findings should be contextualized within Chinese exam-oriented education context, where distinct socio-cultural forces likely amplify the dynamics between self-efficacy, buoyancy, and burnout. The pervasive pressure of high-stakes testing intensifies the psychological consequences of academic setbacks, making self-efficacy a more critical buffer against burnout than in less punitive environments. Furthermore, Confucian values that prioritize effort may inadvertently undermine buoyancy by encouraging rigid persistence over adaptive coping strategies. Finally, collectivistic expectations that tie academic achievement to familial honor can inhibit help-seeking—a key buoyant behavior—due to potent fears of “losing face.” Thus, interventions must not only cultivate personal resilience but also navigate these cultural specificities to effectively reduce burnout.

## 5. Conclusions

Through three follow-up studies conducted at four-month intervals, this study systematically revealed the longitudinal mechanism of academic self-efficacy, academic buoyancy, and academic burnout based on a cross-lagged analysis of 906 junior high school students. The core findings reveal that academic self-efficacy and academic buoyancy have a bidirectional relationship, and together they form a psychological resource system that mitigates academic burnout. Academic buoyancy plays a clear mediating role, not only helping to transmit the adverse association effect of academic self-efficacy on burnout, but also helping to explain the mechanism by which burnout is associated with lower self-efficacy. Theoretically, these findings integrate social cognitive theory with resource conservation theory, empirically validate a dynamic cyclical model among the three constructs, and overcome the limitations of previous research that relied on unidirectional causal assumptions. Methodologically, the application of rigorous measurement invariance tests and a multi-wave cross-lagged panel design provides a robust framework for investigating the temporal dynamics of adaptive mechanisms in adolescents. Practically, this study highlights the value of a dual-path intervention targeting academic self-efficacy and academic buoyancy. Synergistic training programs focused on coping with setbacks and enhancing self-efficacy could effectively disrupt the vicious cycle of “academic burnout → low academic self-efficacy.” The implications of these findings are highly practical. Schools, for instance, can leverage this knowledge by integrating buoyancy-building activities into the curriculum, while parents can be guided to foster self-efficacy through supportive parenting practices. Future research should incorporate ecological variables (e.g., home and school environments), develop and evaluate school-based academic buoyancy curriculum systems, and longitudinally track the developmental trajectories of these adaptive processes across different educational stages. Such efforts will provide a stronger empirical foundation for constructing effective adolescent psychological resilience intervention models.

## Figures and Tables

**Table 1:** Testing the equivalence of academic self-efficacy, academic burnout, and academic buoyancy among junior high school students over time.

Variable	Model	*x* ^2^	*df*	CFI	TLI	SRMR	RMSEA Model Comparison		ΔCFI	ΔRMSEA
Academic self-efficacy	M1: Configuration equivalence	4543.75	1461	0.910	0.901	0.049	0.041			
	M2: Weak equivalence	4638.86	1495	0.908	0.902	0.051	0.041	M2-M1	−0.002	0.000
	M3: Strong equivalence	4825.43	1529	0.904	0.899	0.052	0.041	M3-M2	−0.004	0.000
Academic buoyancy	M1: Configuration equivalence	156.99	39	0.981	0.968	0.026	0.049			
	M2: Weak equivalence	166.35	45	0.980	0971	0.029	0.046	M2-M1	−0.001	0.003
	M3: Strong equivalence	198.88	53	0.976	0.970	0.034	0.046	M3-M2	−0.004	0.000
Academic burnout	M1: Configuration equivalence	2932.66	996	0.926	0.917	0.053	0.039			
	M2: Weak equivalence	2985.18	1022	0.925	0.918	0.053	0.039	M2-M1	−0.001	0.000
	M3: Strong equivalence	3139.57	1048	0.921	0.914	0.054	0.040	M3-M2	−0.004	0.001

Note. Following established guidelines (F. F. Chen, 2007), measurement invariance was supported by meeting the criteria of ΔCFI ≤ 0.01 and ΔRMSEA ≤ 0.015. This indicates that imposing more restrictive equality constraints did not substantially worsen model fit.

**Table 2:** Descriptive statistics and correlation analysis of variables (n = 906).

	*M*	*SD*	1	2	3	4	5	6	7	8	9
1	3.00	0.69	1								
2	2.87	0.74	0.59 **	1							
3	2.86	0.75	0.52 **	0.57 **	1						
4	3.12	0.92	0.62 **	0.46 **	0.42 **	1					
5	3.03	0.98	0.41 **	0.59 **	0.44 **	0.44 **	1				
6	3.02	0.98	0.41 **	0.47 **	0.66 **	0.37 **	0.43 **	1			
7	2.38	0.61	−0.36 **	−0.29 **	−0.30 **	−0.40 **	−0.31 **	−0.30 **	1		
8	2.42	0.64	−0.32 **	−0.40 **	−0.36 **	−0.33 **	−0.41 **	−0.33 **	0.62 **	1	
9	2.49	0.63	−0.23 **	−0.23 **	−0.31 **	−0.25 **	−0.28 **	−0.30 **	0.47 **	0.59 **	1

Note: 1 = T1 academic self-efficacy, 2 = T2 academic self-efficacy, 3 = T3 academic self-efficacy, 4 = T1 academic buoyancy, 5 = T2 academic buoyancy, 6 = T3 academic buoyancy, 7 = T1 academic burnout, 8 = T2 Academic Burnout, 9 = T3 Academic Burnout; ** *p* < 0.01.

## Data Availability

The data that support the findings of this study are available from the corresponding author, Licong Ye, upon reasonable request.
